# Group concept mapping conceptualizes high-quality care for long-stay pediatric intensive care unit patients and families

**DOI:** 10.1016/j.jpeds.2022.08.007

**Published:** 2022-08-13

**Authors:** Brian D. Leland, Lucia D. Wocial, Vanessa N. Madrigal, Michelle M. Moon, Cheryl Ramey-Hunt, Jennifer K. Walter, Jennifer D. Baird, Jeffrey D. Edwards

**Affiliations:** 1 Division of Pediatric Critical Care, Department of Pediatrics, Indiana University School of Medicine; 2 Charles Warren Fairbanks Center for Medical Ethics, Indianapolis, IN; 3 John J. Lynch, MD Center for Ethics, MedStar Washington Hospital Center; 4 Division of Critical Care Medicine, Department of Pediatrics, George Washington University School of Medicine and Health Sciences; 5 Pediatric Ethics Program, Children’s National Hospital, Washington, DC; 6 Palliative Care and Symptom Management, Swedish Health Systems, Issaquah, WA; 7 Integrated Care Management, Case Management, and Social Work, Indiana University Health & Riley Hospital for Children, Indianapolis, IN; 8 Department of Pediatrics, University of Pennsylvania Perelman School of Medicine; 9 Department of Medical Ethics and Health Policy, University of Pennsylvania Perelman School of Medicine; 10 Pediatric Advanced Care Team, Justin Michael Ingerman Center for Palliative Care, The Children’s Hospital of Philadelphia, Philadelphia, PA; 11 Center for Pediatric Clinical Effectiveness, The Children’s Hospital of Philadelphia, Philadelphia, PA; 12 Institute for Nursing and Interprofessional Research, Children’s Hospital Los Angeles, CA; 13 Section of Critical Care, Department of Pediatrics, Columbia University Vagelos College of Physician and Surgeons, New York, NY

## Abstract

**Objective:**

To describe and conceptualize high-quality care for long-stay pediatric intensive care unit (PICU) patients using group concept mapping (GCM).

**Study design:**

We convened an expert panel to elucidate domains of high-quality care for this growing patient population for which transitory care models fail to meet their needs. Thirty-one healthcare professionals and 7 parents of patients with previous prolonged PICU hospitalizations comprised a diverse, interprofessional multidisciplinary panel. Participants completed the prompt “For PICU patients and families experiencing prolonged lengths of stay, high quality care from the medical team includes ______”, with unlimited free text responses. Responses were synthesized into individual statements, then panelists sorted them by idea similarity and rated them by perceived importance. Statement analysis using GCM software through *GroupWisdom* generated nonoverlapping clusters representing domains of high-quality care.

**Results:**

Participants submitted 265 prompt responses representing 313 unique ideas, resulting in 78 final statements for sorting and rating. The resultant cluster map best representing the data contained 8 domains: (1) Family-Centered Care and Shared Decision Making, (2) Humanizing the Patient, (3) Clinician Supports and Resources, (4) Multidisciplinary Coordination of Care, (5) Family Well-Being, (6) Anticipatory Guidance and Care Planning, (7) Communication, and (8) Continuity of Care.

**Conclusions:**

GCM empowered a panel of healthcare professionals and parents to explicitly describe and conceptualize high-quality care for patients and families experiencing prolonged PICU stays. This information will aid the effort to address shortcomings of transitory PICU care models.

Even though the majority of patients admitted to a pediatric intensive care unit (PICU) recover from their critical illness quickly and can be safely transferred out or discharged home, a small but growing population experience prolonged stays.^1–9^ These prolonged PICU hospitalizations are associated with new or existing chronic medical conditions and related disability, increased rates of medical errors, higher patient morbidity and mortality, and negative repercussions for families.^10–15^ Typical transitory PICU care models with rotating physicians, nurses, and other team members may sufficiently meet the needs of patients with average/median lengths of stay; however, these models may create additional barriers to streamlined care for long-stay patients and their families. Regular changes in personnel lead to providers’ lack of familiarity with individual patients, fragmentation of care, and often shifting goals and related strategies, as well as variable timelines for plan implementation and/or assessment. Important conversations surrounding treatment plan milestones (eg, tracheostomy/gastrostomy tube placement) or transitions in care goals (eg, treatment limitations, code status changes) may be delayed secondary to limited rapport with or trust in a particular clinician.^16,17^ Furthermore, team member compassion fatigue, burnout, moral distress, and even post-traumatic stress may occur.^18−23^

In response to the shortcomings of existing PICU care models, some institutions have created and implemented practice changes to promote continuity in care, streamline and augment communication, strengthen relationships, and expedite decision making, among other improvements.^24–27^ These initiatives, with what are arguably similar and overlapping goals, have been developed and implemented primarily at the institutional level, in isolation from one another. Furthermore, a paucity of literature describing the overall thematic framing and comprehensive elements of delivering high-quality care for long-stay patients and their families leaves providers and healthcare systems with limited guidance to inform these practice changes. To address this gap in knowledge, a multidisciplinary group of clinicians, healthcare professionals, and parents of children with medical complexity used group concept mapping (GCM) to collaboratively leverage their expertise, experience, and perspectives, along with the existing literature, to conceptualize and specify what high-quality care for this population entails.

## Methods

GCM is a research methodology that uses both qualitative processes and multivariate statistical analyses to illustrate content pertinent to a specific topic.^28^ This GCM exercise was part of a larger effort to broadly elucidate the needs of long-stay patients in PICUs and then to address one of those needs—continuity of care—by establishing consensus guidelines for PICU continuity strategies. This effort was preceded by a comprehensive literature search that sought articles addressing long-stay patients and problems with transitory care, continuity of care, and PICU-related family-centered care. From this search, the research team created a bibliography, organized and cross-referenced by overarching topics, with a general summary of each topic followed by synopsis of each pertinent article with a link to the article.

Two investigators sought participation from a multidisciplinary group of stakeholders from the US and Canada with diverse backgrounds, relevant clinical expertise, and experience with children with medical complexity and prolonged hospitalization. The sex, racial, and geographic diversity of potential participants was intentionally evaluated during the selection process. Thirty-eight individuals were invited and committed to participate. The interprofessional, multidisciplinary panel of healthcare professionals represented pediatric intensive care, palliative care, complex care, neonatology, postacute/chronic care, nursing, social work, case management, child life services, music/art therapy, chaplaincy, language services, research, medical ethics, and hospital administration (Appendix; available at www.jpeds.com). Of the 38 invitees, 7 were parents of children with medical complexity who had prolonged stays in a PICU, none of whom had any affiliation with the authors or other participants. These parents were experienced patient advocates and were affiliated with Family Voices or the Courageous Parents Network; one parent was also a physician. The number of participants was intentional, to allow for sufficiently broad representation for GCM and for the same participants to work together on the subsequent continuity strategy consensus statements (Appendix).

The *GroupWisdom* platform (Concept Systems Inc; http://groupwisdom.tech) used for the project’s GCM exercise is a proprietary web-based software that uses a structured mixed-methods approach combining qualitative strategies (brainstorming, idea sorting, statement ranking, and descriptive analysis) with quantitative methods (multidimensional scaling and cluster analysis) to ultimately generate cluster maps that collectively illustrate themes representing the study data.^29–31^

Prior to GCM, all participants received the previously described bibliography with synopses to serve as a reference and to stimulate idea formulation. The GCM process includes preparation, brainstorming/statement generation, statement sorting and rating, and map creation. As the first step in the GCM process, the investigators created a focus prompt for GCM participants. The research team used an iterative process identifying the most important questions to elucidate crucial elements in the care of long-stay patients in the PICU, then condensed these into a single prompt that would most effectively utilize participants’ expertise. The prompt read “For PICU patients and families experiencing prolonged lengths of stay, high quality care from the medical team includes ___.” Utilizing their own professional expertise and experience, along with the provided bibliography, participants were instructed to complete the prompt with an unlimited number of short, unique, free-text responses. Deidentified statements were visible to all participants, as they were generated to both limit redundancy and stimulate additional ideas. With support from Concept Systems, 2 investigators performed idea synthesis—involving reviewing each statement, splitting multi-idea statements into individual statements, combining and/or eliminating redundant or conceptually similar statements, ensuring relevance to the project focus, and editing for grammar and spelling—of the provided statements. With access to the panel’s original prompt responses, the remaining coauthors reviewed and collectively confirmed the appropriateness, completeness, and accuracy of the synthesized statements.

On completion of brainstorming, participants were provided the newly synthesized statements and asked to sort them into groups according to conceptual similarity of meaning and/or idea. Participants were not limited in the number of sorted groups they created. In addition to sorting the synthesized statements, participants rated each statement by relative “importance,” using a numeric scale from 1 to 5, with 1 reflecting statements of least importance and 5 reflecting statements of greatest importance. Investigators reviewed importance ratings from each participant, excluding participant responses with homogenous ratings for all statements.

After completion of sorting and rating, the investigators performed an analysis of the statements to generate point and cluster maps. Multidimensional scaling resulted in a point map with each synthesized statement shown as a point in 2-dimensional space in a “galaxy” formation, with respective spatial orientation of statements reflecting idea association as collectively determined by the group’s sorting exercise. The investigators confirmed an appropriate stress value of the map, ensuring that spatial orientation between each point (representing one single statement) on the map was accurately illustrated^32^ and thus an appropriate visual representation of the results of the sorting exercise.^33^ Hierarchical cluster analysis of the point maps then used the Ward algorithm to merge like ideas into 3-dimensional maps of nonoverlapping polygons representing collective participant sorting and rating of statements.^33^ Cluster analysis generated 12 unique maps with 4–15 clusters. The investigators selected the cluster map that they believed best illustrated the data results.^33^ The research team then created domain names for each cluster. The domain names conceptualize the aggregate content within each cluster and represent key themes of high-quality care for long-stay patients and their families. Along with presenting the final cluster map, the research team described the domains with a representative statement and provided the 10 most highly rated statements. All statements, with their respective ratings, are provided separately (Table I; available at www.jpeds.com). Because this project did not involve human subjects, as defined by Health and Human Services Regulation 45CFR46,^34^ Institutional Review Board approval was not sought.

## Results

### Statement Generation, Synthesis, Sorting, and Cluster Map Formation

Thirty-seven invited individuals (97%) participated in 1 or more aspects of the GCM exercise. Thirty-three participants submitted brainstorming ideas, 34 completed idea sorting, and 35 provided importance ratings. All parent participants responded for each component of the GCM process. Project participants generated 265 statements through brainstorming, representing 313 individual ideas. Idea synthesis consolidated these into 78 final statements. Participants sorted synthesized statements into varying numbers of groups, ranging from 5 groups to 19 groups. The resulting point map stress value was 0.30, within the 0.20–0.36 recommended range, indicating a good fit for the data set, with consistency between values in the similarity matrix generated from statement sorting and distances displayed on the point map.^32,33^

The cluster map that the research team collectively agreed best represented the data contains 8 nonoverlapping clusters (Figure): (1) Family-Centered Care and Shared Decision Making, (2) Humanizing the Patient, (3) Clinician Supports and Resources, (4) Multidisciplinary Coordination of Care, (5) Family Well-Being, (6) Anticipatory Guidance and Care Planning, (7) Communication, and (8) Continuity of Care. These domains are described with representative statements in Table II.

### Importance Rating

As described above, project participants rated the relative importance of each synthesized statement. One participant’s ratings were excluded owing to a lack of variability when rating respective statement importance. The top-10 rated statements and a comprehensive list of statements with their corresponding average importance rating, grouped by domain, are provided in Table III. Two domains collectively contained the 10 highest-rated statements. Domain 2, Humanizing the Patient, contained one top-10 statement, “Respecting the patient as a unique individual deserving compassion and kindness,” which tied for the highest average statement importance rating, at 4.83 out of 5. Domain 1, Family-Centered Care and Shared Decision Making, contained the remaining 9 top-10 statements, with average statement importance ratings ranging from 4.64 to 4.83. The 8 respective domains varied in average aggregate statement importance rating, with Domains 1 and 2 carrying the 2 highest average aggregate importance ratings at 4.47 and 4.22, respectively, and Domain 7, Communication, and Domain 8, Continuity of Care, tied for the lowest average aggregate statement importance rating at 3.86 (Table II).

## Discussion

This GCM exercise resulted in a multidisciplinary, expert-informed framework conceptualizing and specifying high-quality care for PICU patients and their families with prolonged length of stay. Health outcomes for children with medical complexity have been explored through GCM^35^; this study leveraged GCM to frame high-quality care for this subpopulation specifically. Similarly, although guidelines for general family-centered PICU care are applicable to long-stay patients and their families, the use of GCM complements those recommendations by narrowing the focus on this uniquely vulnerable pediatric population.^36,37^ GCM goes beyond commentaries from a single expert or a few experts by synthesizing the experiences and expertise of a diverse group of key stakeholders, including family members with lived experience.

The generated cluster map empowers health care teams, hospitals, and health care systems to prioritize initiatives and programmatic changes that address opportunities to provide high-quality care. Eight domains are established containing explicit statements that collectively conceptualize themes of high-quality care for patients and families experiencing prolonged PICU stays. The most highly rated statements focus on relationship building, burden sharing, and proactive planning informed by patient and family values (patient/family-centered care), as opposed to statements representing medical expertise or delivery of information. The 2 domains with the highest average aggregate importance ratings were Family Centered Care and Shared Decision Making (Domain 1) and Humanizing the Patient (Domain 2). Domain 1 contained not only the highest average statement rating at 4.47, but also the highest number of statements of any domain at 20, including “realistic discussions of the patient’s/family’s goals of care in the context of expected prognosis” and “patients/families feeling and being heard,” among others.

Notably, the results illustrate and reinforce that prolonged PICU admission impacts not only patients and families, but also institutional resources and personnel. Domain 3, Clinician Supports and Resources, emphasizes that high-quality care for long-stay patients and their families requires support mechanisms for the PICU team. This domain contained the representative statements “regular assessment of the emotional wellbeing of providers” and “professional caregivers empowered with tools and resources to mitigate stressors.,” among others. Potential candidates for team support mechanisms include nursing team rotations for long-stay patients, compassion rounds for exhausted providers, scheduled and as-needed check-ins by leadership of respective disciplines, and resiliency training for team members,^38^ among others. The literature describes PICU team members as being at risk of moral distress, compassion fatigue, and becoming jaded as they care for long-stay patients in challenging circumstances.^18–21,23,36,37^ Considering that moral distress is a positive predictor for leaving a profession and the unprecedented nursing shortage nationally, renewed efforts to promote health care team member well-being and thus the highest-quality patient care merit serious consideration.

Although evidence suggests that initiatives promoting continuity for long-stay patients may improve family satisfaction, mitigate negative family affect, and shorten length of hospitalization, they have an overwhelming focus on inpatient outcomes.^26,39–41^ In contrast, this study’s data and related cluster map draws attention to the inseparability of in-hospital and out-of-hospital dynamics and the coordination challenges that long-stay patients must navigate. Several statements illustrate this phenomenon, including but not limited to “engaging the patient’s primary care provider and longstanding subspecialists in important decisions” and “preparing families with the knowledge, skills, equipment, appointments, and providers needed to care for their child after transfer out of the PICU.” It is revealing then that most, if not all, of the 8 cluster domains describe constructs that are not necessarily isolated to the PICU or even to the inpatient hospital setting as a whole. This suggests that during prolonged PICU hospitalizations, treatment plans and decisions should not be framed as isolated events or siloed from the rest of the patient’s care overall. High-quality care encompasses planning for potential impact to the home environment, communication and partnerships with outpatient providers, attention to PICU team member well-being, and respite for exhausted parents.

For units and institutions wishing to use these study results to inform practice changes to improve the care of long-stay patients and their families, prioritizing efforts can be challenging. Even though there exists a hierarchy in the importance ratings of both individual statements (Tables I and III) and average importance ratings of respective cluster domain statements (Figure,Table II), the differences in absolute values are modest, with the first and tenth top individual statement importance ratings differing by only 0.19 (4.83 and 4.64, respectively) and the first and eighth cluster domain importance ratings differing by 0.61 (4.47 and 3.86, respectively). Acknowledging minimal differences, we recommend that institutions and healthcare teams adopt a multifaceted and personalized approach to prioritization, matching self-identified needs/barriers of a respective center with the cluster domains with the highest yield and most feasible foci to address. It is clear that all domains and their respective aggregate statements are of substantial importance to the panel responsible for this GCM effort, and the sentiment that there is a “wrong choice” for prioritization because of a slightly lower importance rating should be discouraged. We do suggest that Domain 1, Family-Centered Care and Shared Decision Making, with both the highest domain importance rating and 9 of the 10 highest individual statement ratings, be weighed heavily in any potential practice change, and that providers individually and collectively recommit themselves to compassionate, conscientious patient/family-centered care.^36,37^ Many of the statements contained in Domain 1 describe opportunities for setting expectations, establishing roles, and placing decisions in the context of family values. Additional considerations for institutions/PICUs could include enhancing partnerships with families and implementing primary physicians and nursing teams to promote relationship-building and communication and streamline consistency in care delivery.^38^

This study has several limitations. First, aspects of the GCM process are necessarily subjective, requiring members of the research team to use their expert judgment and an understanding of the project goals to guide specific decisions. For example, the research team excluded one respondent’s importance ratings owing to a lack of rating variability, and 8 domains were chosen for our final cluster map, as we agreed that those specific cluster domains best represent the project’s findings. It is reasonable that a different group of researchers might have selected a final cluster map with a different number of clusters. Second, the total GCM participant group was relatively small compared with that in some other GCM projects. It is possible that a larger number of participants might have elucidated additional constructs pertinent to the care of long-stay patients. However, given the range of disciplines and perspectives represented, there was collective agreement that the panel was appropriately representative. Moreover, the investigators agreed that it was advantageous to have the same participants work together through GCM, and then the subsequent consensus guidelines, as a larger number of participants would have resulted in a considerably less manageable, and perhaps less fruitful, consensus effort. Third, rating of synthesized statements is inherently subjective, and personal experience as well as professional discipline might have biased statement ratings. This also could be viewed as a strength, as the broad representation and diversity of participants likely muted biases of any particular individual. Fourth, participation in each component involved in the GCM process was incomplete, with some failing to either submit brainstorming, perform idea sorting, or assign importance ratings to statements. This could have introduced bias depending on which participants were not represented. Fortunately, with 89% of participants completing brainstorming, 92% completing idea sorting, and 95% completing statement importance rating, the impact of nonparticipants should have been minimized. Finally, the GCM methodology itself is complicated and nuanced; for example, some statements may seem incongruent in their respective domain. Statement 62, “allowing nurses who consistently care for the same patient over weeks/months an opportunity to take care of other patients in order to maintain their skills and/or avoid burnout,” appears out of place in Domain 2, Multidisciplinary Coordination of Care; however, the location of statement 62 is not because of its close relationship to other statements in its cluster, but rather because of its broader relationship to other statements remote to it on the map (ie, its high bridging value).^33^

Existing models of care tend to best support short stays, leaving patients and families enduring prolonged PICU admissions negatively impacted and their care suboptimal. In this study, GCM empowered a multidisciplinary group of medical experts and family members to conceptualize and specify what is high-quality care for these long-stay patients and their families. The resultant cluster map presents a framework for optimal care, which can inform initiatives and policies to better meet the needs of this inherently vulnerable population and address shortcomings of the transitory nature of traditional PICU care models.

## Supplementary Material

Appendix 1

## Figures and Tables

**Figure.**
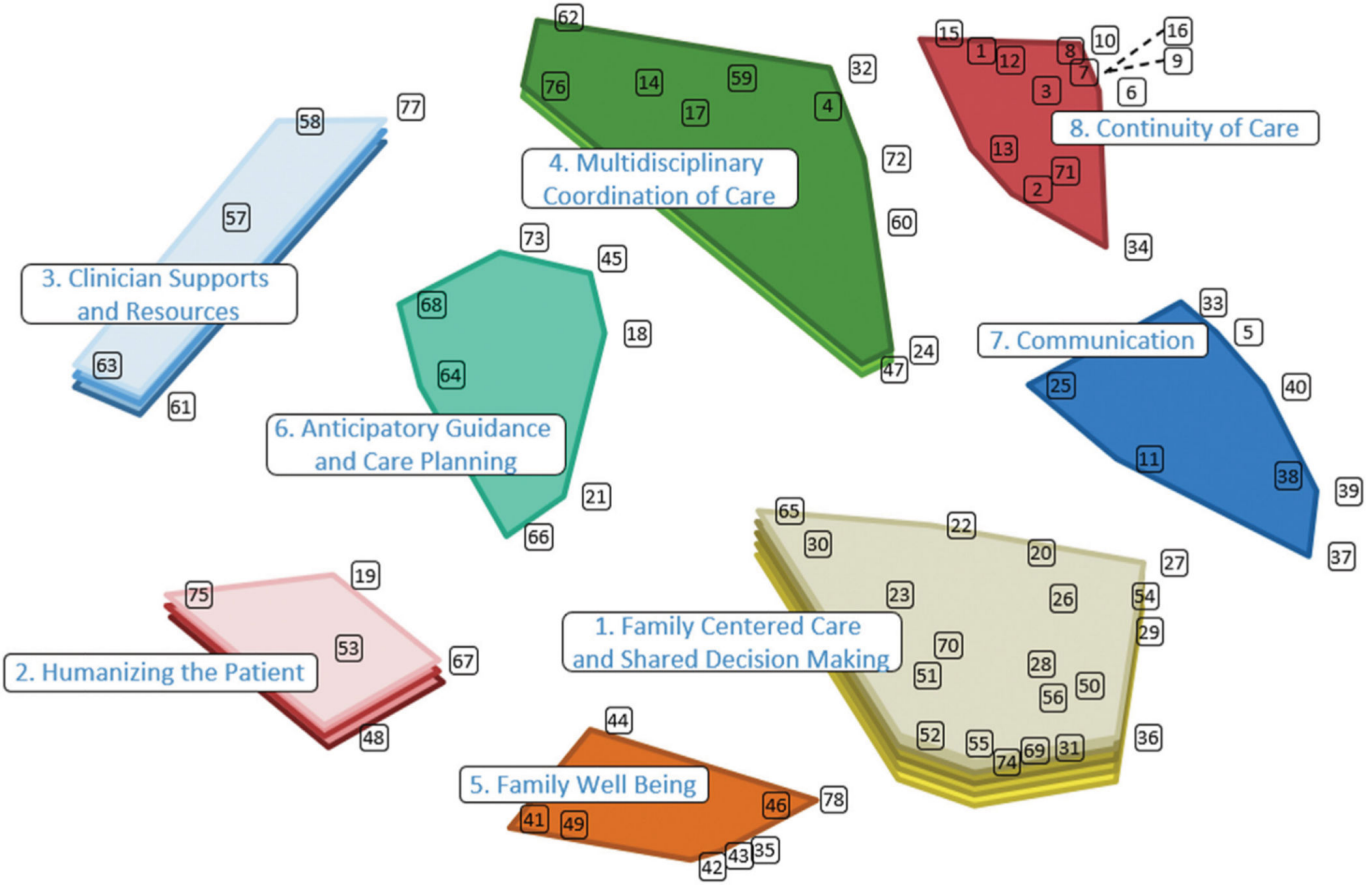
Final cluster map illustrating 8 domains of high-quality care for long-stay PICU patients and families, respective statement numbers contained within each domain, and respective cluster importance layering.

**Table I.** Top 10 synthesized statements by importance rating

Average importance rating	Statement no./domain name (no.)	“For PICU patients and families experiencing prolonged lengths of stay, high quality care from the medical team includes ______”
4.83	53/Humanizing the Patient (2)	Respecting the patient as a unique individual deserving compassion and kindness
4.83	26/Family-Centered Care and Shared Decision Making (1)	Ensuring families have the information they need to make an informed decision about their child’s care
4.77	M4/Family-Centered Care and Shared Decision Making (1)	A healthy, mutually respectful relationship between the patient/family and medical team
4.71	28/Family-Centered Care and Shared Decision Making (1)	Facilitating communication to the patient/family in their language of choice or in a way that meets their needs (nonverbal, at their health literacy level, etc)
4.71	31/Family-Centered Care and Shared Decision Making (1)	Patients/families feeling and being heard
4.71	56/Family-Centered Care and Shared Decision Making (1)	A collective understanding of and respect for the patient’s/family’s perspectives, values, and goals of care
4.69	22/Family-Centered Care and Shared Decision Making (1)	Realistic discussion of the patient’s/family’s goals of care in the context of expected prognosis
4.69	69/Family-Centered Care and Shared Decision Making (1)	Recognizing families as “experts” with regard to their child
4.66	30/Family-Centered Care and Shared Decision Making (1)	Willingness and ability to have sincere, candid, empathetic, timely, and balanced conversations about difficult topics (eg, goals of care, end of life, code status)
4.64	52/Family-Centered Care and Shared Decision Making (1)	Allowing/encouraging family involvement in the bedside care of the patient

**Table II.** Cluster domains with synthesized statements, comprehensive

Domain number and name	Domain statement	Importance rating (domain)/statement
1. Family-Centered Care and Shared Decision Making		(4.47)
	26. Ensuring families have the information they need to make informed decisions about their child’s care	4.83
	54. A healthy, mutually respectful relationship between the patient/family and medical team	4.77
	28. Facilitating communication with the patient/family in their language of choice or in a way that meets their needs (nonverbal, at their health literacy level, etc)	4.71
	31. Patients/families feeling and being heard	4.71
	56. A collective understanding of and respect for the patient’s/family’s perspectives, values, and goals of care	4.71
	22. Realistic discussions of the patient’s/family’s goals of care in the context of expected prognosis	4.69
	69. Recognizing families as “experts” with regard to their child	4.69
	30. Willingness and ability to have sincere, candid, empathetic, timely, and balanced conversations about difficult topics (goals of care, end of life, code status, etc)	4.66
	52. Allowing/encouraging family involvement in the bedside care of the patient	4.64
	65. Realistic two-way discussions about the patient’s illness trajectory and associated current and projected quality of life	4.63
	70. Alignment of perspectives and goals of patients/families and the medical team	4.49
	23. Putting all clinical decisions in the context of the patient’s/family’s goals and values	4.46
	55. A collective understanding of and respect for the patient/family dynamics/culture/religion/etc	4.44
	27. Means to ensure effective communication with families when they are not able to be at the bedside	4.43
	20. Facilitating mutual understanding and agreement of goals and care plans among surrogate decision makers	4.34
	74. Supporting families, whenever possible, in their determinations of the “best interests” of their child while recognizing there can be divergent opinions among parties	4.33
	51. Patience from the medical team (to make decisions, absorb information, etc)	4.29
	50. Assurance from the medical team that patients/families Will not be abandoned	4.17
	29. Daily engagement with the patient/family	4.11
	36. Offering family-to-family support resources	3.49
2. Humanizing the Patient		(4.22)
	53. Respecting the patient as a unique individual, deserving compassion and kindness	4.83
	19. Consistent efforts to minimize all types of suffering (physical, emotional, spiritual, etc)	4.60
	67. Preparing families with the knowledge, skills, equipment, appointments, and providers needed to care for their child after transfer out of the PICU	4.57
	75. Developmentally appropriate nonmedical activities	3.67
	48. Treating the intensive care unit room as the child’s “bedroom” to promote a sense of comfort and safety	3.40
3. Clinician Supports and Resources		(4.10)
	77. Evidence-based practices to avoid hospital- acquired complications	4.31
	58. Education/training/competence for PICU providers on complex chronic conditions and care	4.19
	57. Education/training/competence in “primary” palliative care (ie, basic knowledge and skills for symptom management, clear and sensitive communication, and shared decision making based on patients’ values, goals, and preferences)	4.09
	61. Professional caregivers empowered with tools and resources to mitigate stressors experienced in the care of this patient population, such as moral distress, compassion fatigue, and burnout	4.08
	63. Regular assessment of the emotional well-being of providers	3.78
4. Multidisciplinary Coordination of Care		(4.06)
	59. Providers who are knowledgeable in the patient’s underlying and acute conditions	4.63
	24. Ensuring all providers know the patient’s/family’s goals of care and advance directives	4.54
	72. Multidisciplinary care	4.39
	60. A team environment where members are encouraged to openly communicate concerns about approaches to patient management	4.37
	4. A collective “memory” of successful and unsuccessful management approaches for the patient	4.17
	62. Allowing nurses who consistently care for the same patient over weeks/months an opportunity to take care of other patients to maintain their skills and/or avoid burnout	3.97
	32. Regular engagement with the patient’s important outpatient providers, especially around the time of transfer out of the PICU	3.92
	17. Clear criteria for when strategies for long-stay patients should be initiated	3.86
	47. An accessible means to display the patient’s short- and long-term care goals, daily routine, and other important information, developed with the help of the patient/family	3.72
	14. Accountability for follow-up/follow-through on nonemergent care needs	3.51
	76. Following management changes with a deliberate evaluation period, in nonurgent situations, to assess their effect	3.49
5. Family Well-Being		(3.92)
	46. Safe spaces (physical and emotional) for patients/families to be able to share their worries, fears, and hopes without judgment	4.46
	78. Acknowledging that caring for the patient and caring for the family are mutually inclusive	4.17
	35. Effort and resources to support families experiencing or at risk for adverse psychological/social/financial stressors, regardless of whether they stem from inside or outside the hospital	4.06
	41. An environment amenable, as possible, for sleep/rest, remote working, and self-care	3.94
	42. Opportunities for families to focus on their well-being (eg, respite)	3.89
	44. Trauma informed care (ie, recognizing and validating the effects of prior events, especially psychosocial trauma, on patients/families to create an environment of safety, empowerment, and healing)	3.83
	43. Regular assessments of the emotional well-being of family members	3.69
	49. Specialists/resources to support postpartum mothers	3.36
6. Anticipatory Guidance and Care Planning		(3.90)
	21. The option to withdraw life-sustaining interventions, whether in the hospital or at home, with appropriate support in terminally ill patients	4.43
	68. Anticipatory guidance to patients/families on transitions out of the PICU (eg, criteria for safe transfer, postdischarge challenges, resources needed)	4.11
	66. Creating/updating the patient’s emergency plan with family prior to discharge/transfer	3.94
	18. Proactive and recurring engagement with palliative care services	3.91
	73. Referrals/second opinions out of the institution if the child’s medical needs are too complex	3.83
	45. Consistent access to child life services and education specialists	3.63
	64. Anticipatory guidance on the morbidity and mortality associated with prolonged PICU stays	3.57
7. Communication		(3.86)
	33. Consistent, synthesized messages and recommendations from the medical team	4.50
	11. Regular multidisciplinary meetings with the patient/family	4.22
	25. Clearly communicated expectations and roles of all involved parties	4.20
	38. Consistent access to social work and related resources	4.08
	40. Consistent access to a family navigator/liaison and related specialists/resources	3.53
	39. Consistent access to psychologists and related resources	3.51
	37. Consistent access to chaplaincy services	3.47
	5. The ability for the patient/family to request a “primary attending” or similar person, even when they do not meet the unit’s normal criteria for assigning one	3.34
8. Continuity of Care		(3.86)
	34. Effective communication and information sharing among all the medical team members	4.63
	15. A sustainable approach to continuity of care	4.34
	1. Consistency in how care is provided from shift to shift, week to week, person to person, etc	4.25
	2. Shared approaches, goals, and timelines among the supervisory doctors	4.20
	6. Continuity of nurses (or group of “primary nurses”)	4.14
	12. The PICU team taking responsibility for providing continuity, even if other services follow the patient	3.97
	13. Engaging the patient’s primary care provider and longstanding subspecialists in important decisions	3.97
	3. A supervisory doctor (or “primary attending”) who is designated to facilitate informational, management, and relational continuity	3.91
	9. Continuity of frontline providers (residents, nurse practitioners, physician assistants, etc)	3.89
	7. Continuity providers who are drawn from both day and night shifts	3.58
	16. Continuity of subspecialty consultants	3.53
	71. Inclusion of subspecialists on daily rounds, as feasible	3.34
	8. Continuity of therapists (respiratory therapy, physical therapy, occupational therapy, child life specialists, music/art therapists, etc)	3.31
	10. Continuity of child life specialists	3.06

**Table III.** GCM domain name, description, and representative statement

GCM domain (average importance rating)	Domain description	Representative synthesized statement*
1. Family-Centered Care and Shared Decision Making (4.47)	Care informed by patient and family values and mutually established goals	Supporting families, whenever possible, in their determinations of the “best interest” of their child while recognizing there can be divergent opinions among parties
2. Humanizing the Patient (4.22)	Maintaining a holistic approach to patient care and promoting human well-being and dignity	Developmentally appropriate nonmedical activities
3. Clinician Supports and Resources (4.10)	Infrastructure to mitigate the effects of stressors associated with care of long-stay patients in team members, and education and tools to facilitate care	Professional caregivers empowered with tools and resources to mitigate stressors experienced in the care of this patient population, such as moral distress, compassion fatigue, and burnout
4. Multidisciplinary Coordination of Care (4.06)	Plans that reflect the collective input from all disciplines integral to the patient’s care	Ensuring all providers know the patient’s/family’s goals of care and advance directives
5. Family Well-Being (3.92)	Care that reflects a recognition of trauma and stressors experienced by families of patients with prolonged and often recurring hospitalizations	Trauma-informed care, recognizing and validating the effects of prior events, especially psychosocial trauma, on patients/families to create an environment of safety, empowerment, and healing
6. Anticipatory Guidance and Care Planning (3.90)	Proactive communication, preparation, education, and training to empower decisions and successful transitions in care	Anticipatory guidance to patients/families on transitions out of the PICU (eg, criteria for safe transfer, postdischarge challenges, resources needed)
7. Communication (3.86)	Recurring, honest, transparent, bidirectional information sharing within the medical team and between team and patient/family	Consistent synthesized messages and recommendations from the medical team
8. Continuity of Care (3.86)	Care models that are intentionally longitudinal, limiting fragmentation	Continuity of frontline providers (residents, nurse practitioners, physician assistants, etc)

* Statement prompt: “For PICU patients and families experiencing prolonged lengths of stay, high-quality care from the medical team Includes ______.”

## Abbreviations

GCM: Group concept mapping
PICU: Pediatric intensive care unit
