# Supplementary material for: Group concept mapping conceptualizes high-quality care for long-stay pediatric intensive care unit patients and families
Source: J Pediatr. Author manuscript; Available in PMC 2024 Jan 1. (PMC9772094; doi:10.1016/j.jpeds.2022.08.007)
Supplement: Appendix 1 [file NIHMS1833781-supplement-Appendix_1.docx]

Appendix 1: GCM Participant Name, Credentials, and Institution.

Jennifer Akers

Mother of a child with medical complexity

Former Program Director, Family Voices of Indiana

Jennifer Baird, PhD, MPH, MSW, RN, CPN

Director, Institute for Nursing and Interprofessional Research, Children’s Hospital Los Angeles

Former PICU nurse

Sarah Carlson

Mother of a child with medical complexity

Director of 4 intermediate care facilities/homes for adults with disabilities

Family Consultant, Family Voices of North Dakota

Experienced Parent, Early Intervention Program, Western North Dakota

Franco Carnevale, RN, PhD, PhD

Professor of Nursing, McGill University

Founder and principal investigator, Views On Interdisciplinary Childhood Ethics

Associate Member of Pediatric Critical Care, Montreal Children’s Hospital

Jennifer Check, MD, MS

Associate Professor of Pediatrics, Wake Forest School of Medicine

Medical Director, NICU Follow Up Clinic, Wake Forest Baptist Health - Brenner Children's Hospital

Co-Director, Comprehensive Infant Clinic and Care Collaboration for Babies with Extended Stays

(CCBES)

Attending neonatologist, Wake Forest Baptist Health - Brenner Children's Hospital

Erin Chávez

Manager, Language Services

Indiana University Health

Anne-Marie Cirrilla, MSN, RN

Former manager, Care Coordination and Social Work, New York-Presbyterian Morgan Stanley Children’s Hospital

Former PICU nurse

Karen Cook, PhD, MSc, BSN

Faculty of Health Disciplines, Athabasca University, Athabasca, Alberta, Canada

School of Rehabilitation therapy, Queen’s University, Kingston, Ontario, Canada

Timothy Corden, MD

Kaemmer Professor of Pediatrics, Medical College of Wisconsin

Vice-Chair of Population Health, Medical College of Wisconsin

Section Chief, Medical Director, Special Needs Program, Children’s Hospital of Wisconsin

Former pediatric intensivist

Heather Crowley

Mother of a child with medical complexity

Family Peer Specialist, Missouri Family to Family

Aaron DeWitt, MD

Assistant Professor of Clinical Pediatrics, University of Pennsylvania Perelman School of Medicine

Attending Cardiac ICU physician, Children’s Hospital of Philadelphia

Jeffrey Edwards, MD, MA, MAS

Associate Professor of Pediatrics, Columbia University Medical Center

Medical Director, Medical Surgical Pediatric Intensive Care Unit, Morgan Stanley Children’s Hospital

Attending PICU physician, Morgan Stanley Children’s Hospital

Xiomara Garcia, MD

Associate Professor of Pediatrics, College of Medicine, University of Arkansas

Director, Cardiovascular ICU Chronic Care Team, Arkansas Children’s Hospital

Attending Cardiac ICU physician, Arkansas Children’s Hospital

Denise Goodman, MD, MS

Professor of Pediatrics (Critical Care), Northwestern University, Feinberg School of Medicine

Attending PICU physician, Ann & Robert H. Lurie Children's Hospital of Chicago

Jeanine Graf, MD

Associate Professor of Pediatrics, Baylor College of Medicine

Chief Medical Officer, Texas Children’s Hospital West Campus

Former Medical Director of the PICU and Respiratory Care, Texas Children’s Hospital

Attending PICU physician, Texas Children’s Hospital

Robert Graham, MD

Associate Professor of Anesthesia, Harvard Medical School

Director, Critical Care, Anesthesia, Perioperative, Extension and Home Ventilation Program

Attending PICU physician, Boston Children’s Hospital

Daniel Grossoehme, DMin, MS, BCC

Associate Research Scientist, Haslinger Family Pediatric Palliative Care Center, Akron Children’s Hospital

Former Staff Chaplain III & Assistant Professor of Pediatrics (Research), Cincinnati Children’s Hospital

Past President, Assembly of Episcopal Healthcare Chaplains

Scott Hagen, MD

Associate Professor of Pediatrics, University of Wisconsin School of Medicine and Public Health

Medical Director, PICU, American Family Children’s Hospital

Ann Hannan, MT-BC

Director, Riley Cheer Guild and Music Therapy

Riley Hospital for Children at Indiana University Health

Debbi Harris, MA, MS, GCAS-Creative Writing/Narrative Medicine

Mother of a young man with medical complexity

Systems Specialist, Family Voices of Minnesota

Catherine Haut, DNP, CPNP-AC, PC, FAANP

Coordinator of Nursing Research and Evidence Based Practice

Nemours AI duPont Hospital for Children

Tara Hayes, BS, CCHW

Mother of a child with medical complexity

Family Voices manager, Rhode Island Parent Information Network

Shawnda Hicks

Mother of a child with medical complexity

NICU and PICU family navigator for families in rural and tribal communities

Family Leader, Family-to-Family Health Information Center, Family Voices of Washington

Michael Johnson, MD

Chair, Clinical Practice & Research, Pediatric Complex Care Association

Board member, Pediatric Complex Care Association

Vice President of Medical Services & Medical Director, The Children’s Center Rehabilitation

Hospital

K. Jane Lee, MD, MA

Associate Professor of Pediatrics and Bioethics & Medical Humanities, Medical College of Wisconsin

Attending physician, Special Needs Program and PICU, Children’s Hospital of Wisconsin

Brian Leland, MD, FAAP

Associate Professor of Pediatrics, Indiana University School of Medicine

Director, Charles Warren Fairbanks Center for Medical Ethics

Medical Director for Pediatric Ethics, Riley Hospital for Children

Fellowship Director, Fairbanks Center for Medical Ethics

Attending PICU physician, Riley Hospital for Children

Vanessa Madrigal, MD, MSCE

Associate Professor of Pediatrics, The George Washington University

Director, Pediatric Ethics Program, Children’s National Medical Center

Attending PICU physician, Children’s National Medical Center

Michelle Moon, DO

Mother of a child with medical complexity

Attending palliative care physician, Swedish Health Systems, Issaquah campus

Courageous Parents Network contributor

Becky Parlow, BSN, RN, CCRN-K

PICU Nurse Manager, Nemours AI duPont Hospital for Children

Cheryl Ramey-Hunt, MSW, LCSW

Director, Integrated Care Management, Case Management, and Social Work, Indiana University

Health & Riley Hospital for Children

Lynn Roberts, RN, BSN, CCRN

PICU nurse, Lucile Packard Children’s Hospital

Former nurse, Chronically Critically Ill team, Children’s Hospital of Wisconsin.

Marilyn Sanders, MD

Professor of Pediatrics, University of Connecticut School of Medicine

Director, Connecticut Perinatal Quality Collaborative

Member, Ethics Committee, Connecticut Children’s Hospital

Attending neonatologist, Connecticut Children’s Hospital

Christine Schindler, PhD, RN, CPNP-AC/PC, WCC

Clinical Associate Professor, Marquette University

APP Director for Critical Care/Palliative Care at Medical College of Wisconsin/Children’s Hospital

of Wisconsin

Former PICU and Special Needs nurse practitioner, Children’s Hospital of Wisconsin

Dawn Schwartz, DNP, ARNP, NNP-BC, IBCLC, CBIS, CPN, CHPPN, CCFP, CENP, CPHQ

Vice President of Care Quality, ChildServe

Former Director of Nursing and Pediatric Palliative Care Consultant, ChildServe

Chair, Clinical Practice & Research Committee, Pediatric Complex Care Association

Member of Education Committee, Pediatric Complex Care Association

Katherine Steffen, MD

Clinical Associate Professor of Pediatrics, Stanford University School of Medicine

Member, Stanford Maternal & Child Health Research Institute (MCHRI)

Physician lead for PICU Local Improvement Team and Associate Medical Director of Quality,

Lucile Packard Children’s Hospital

Attending PICU physician, Lucile Packard Children’s Hospital

Jennifer Walter, MD, PhD, MS

Associate Professor of Pediatrics and Medical Ethics and Health Policy, University of Pennsylvania Perelman School of Medicine

Core faculty, Center for Pediatric Clinical Effectiveness and PolicyLab, The Children’s Hospital of

Philadelphia

Attending physician, Justin Michael Ingerman Center for Palliative Care, The Children’s Hospital of Philadelphia

Lucia Wocial, PhD, RN, FAAN, HCE-C

Adjunct Assistant Professor of Nursing, Indiana University

Program Leader for Nursing Ethics, Charles Warren Fairbanks Center for Medical Ethics

Nurse Ethicist, Indiana University Health
